# Comparing intraoperative radiotherapy (IORT) and hypofractionated stereotactic radiotherapy (HSRT) after brain metastasis surgery: impact on oncological outcome and radionecrosis

**DOI:** 10.1007/s11060-025-05152-4

**Published:** 2025-08-13

**Authors:** Maria Neu, Ehab Shiban, Philipp Krauss, Björn Sommer, Zoha Roushan, Susanne Gutser, Christoph J. Maurer, Tilman Janzen, Georg Stüben, Klaus-Henning Kahl

**Affiliations:** 1 Department of Radiotherapy and Radiation Oncology, Faculty of Medicine, University of Augsburg, 86156 Augsburg, Germany; 2 Comprehensive Cancer Center Augsburg (CCCA), Faculty of Medicine, University of Augsburg, 86156 Augsburg, Germany; 3 Comprehensive Cancer Center Alliance WERA (CCC WERA), 86156 Augsburg, Germany; 4 Bavarian Cancer Research Center (BZKF), 86156 Augsburg, Germany; 5 Department of Neurosurgery, Lausitz University Hospital, Cottbus, Germany; 6 Department of Neurosurgery, Faculty of Medicine, University of Augsburg, 86156 Augsburg, Germany; 7 Department of Radiation Protection and Medical Physics, Faculty of Medicine, University of Augsburg, 86156 Augsburg, Germany; 8 Department of Diagnostic and Interventional Neuroradiology, Faculty of Medicine, University of Augsburg, 86156 Augsburg, Germany; 9 Klinik für Strahlentherapie und Radioonkologie, Universitätsklinikum Augsburg, Steglinstr.2, D86156, Augsburg, Germany

**Keywords:** Brain metastases, Surgery, IORT, Hypofractionated stereotactic radiotherapy, Local control, Radionecrosis, Cavity radiotherapy, Focal radiotherapy

## Abstract

**Purpose:**

Due to significantly lower neurocognitive toxicity, whole-brain irradiation (WBI) has largely been replaced by focal irradiation of the resection cavity following brain metastasis surgery. However, the optimal treatment modality and fractionation scheme remain controversial. This study conducts a comparative analysis of hypofractionated stereotactic radiotherapy (HSRT) and intraoperative radiotherapy (IORT), focusing on clinical outcomes and toxicity profiles.

**Methods:**

A retrospective cohort study was conducted, analyzing 129 patients (HSRT: 72, IORT: 57) with 137 treated cavities (HSRT: 75, IORT: 62) at the University Hospital of Augsburg (UKA) between 2013 and 2021. Baseline characteristics, oncological outcomes, incidence of radionecrosis (RN), and time to further treatment were compared.

**Results:**

Radionecrosis occurred significantly less frequently in the IORT group compared to HSRT, with 1-year RN rates of 3.7% (95% CI: 0.5–23.5%) and 21.8% (95% CI: 11.7–39.2%), respectively (*p* = 0.00025). At two years, the RN rate remained substantially lower after IORT (8.5% vs. 53.2%). Notably, in patients without prior cerebral irradiation, no symptomatic RN (sRN) occurred following IORT, whereas the 2-year sRN rate in the HSRT group reached 35.5% (*p* = 0.0036). Oncological outcomes, including overall survival (OS), local control (LC), intracranial disease control, leptomeningeal dissemination (LMD), and WBI avoidance, were comparable between the two groups. However, distant brain control (DBC) at one year was higher in the HSRT group. While HSRT was initiated after a median delay of 29 days (range: 14–71), IORT was delivered intraoperatively, enabling immediate continuation of systemic therapy.

**Conclusion:**

In this retrospective single-center analysis, IORT demonstrated comparable oncological efficacy to HSRT while significantly reducing the risk of RN. Given its intraoperative delivery and the ability to promptly resume systemic therapy, and the precise application directly at the resection cavity, IORT may represent a practical and effective alternative in selected patients.

## Introduction

Surgical resection is an effective treatment for large or symptomatic brain metastases, providing rapid symptom relief and reducing mass effect. However, without adjuvant therapy, local recurrence rates can reach up to 85% [1]. While WBI has historically been used to reduce both local and distant brain failure, its impact on OS is limited, and its use has declined due to neurocognitive toxicity [2]. To improve LC while minimizing neurocognitive toxicity, stereotactic radiotherapy has become the standard approach for post-resection cavity irradiation. Among focal radiotherapy modalities, HSRT is well established, allowing precise dose delivery while sparing healthy brain tissue. IORT has emerged as an alternative, enabling immediate radiation delivery during surgery, potentially mitigating delays associated with external beam radiotherapy (EBRT) and reducing uncertainties in target volume delineation.

With advances in systemic therapy, patients with stage IV malignancies, including melanoma, colorectal cancer (CRC), and non-small cell lung cancer (NSCLC), are experiencing prolonged survival, necessitating treatment strategies that consider long-term toxicity and interactions with systemic therapy. Given that most patients with brain metastases require systemic treatment for extracranial disease, selecting a radiation modality that optimizes LC while minimizing neurotoxicity is increasingly relevant.

This study builds on previous reports of IORT outcomes, including an initial analysis of 40 patients and an expanded cohort of 117 procedures [3, 4]. Here, we provide a direct comparison of IORT and HSRT in terms of oncological outcomes and treatment-related toxicity, offering further insight into their respective roles in the management of resected brain metastases.

## Materials and methods

### Study design and participants

This retrospective analysis included patients who underwent resection of brain metastases at UKA between January 2013 and November 2021 and subsequently received either IORT or HSRT as adjuvant cavity irradiation. The time point for the last follow-up included in this analysis was October 18th, 2023. Eligibility required a minimum distance of 5 mm between the resection cavity and critical structures such as the optic tract or brainstem. Treatment decisions were based on recommendations from the multidisciplinary tumor board (MTB).

### Treatment protocols

IORT was administered intraoperatively following confirmation of malignancy via frozen section analysis. A spherical applicator was positioned by the neurosurgeon to ensure optimal coverage of the resection cavity. Radiation was delivered using the INTRABEAM system (ZEISS MEDITEC AG, Oberkochen, Germany), emitting 50 kV X-rays. The dose was prescribed to the applicator surface, consistent with the target volume concept of postoperative stereotactic radiosurgery (SRS) cavity treatment: Gross target volume (GTV) = clinical target volume (CTV) = cavity. The prescribed surface dose was 20 Gy (median: 20 Gy, range: 13.4–30 Gy). Due to the immediate collapse of the cavities after resection the applicator size was smaller than the preoperative size of the metastasis The median diameter of the used spherical applicators in the IORT group was 2.0 cm (range 1.5–4.0 cm) The dosimetric characteristics of the system, including dose distribution at various depths, have been previously reported and align with prior institutional experience [4–6].

For patients receiving HSRT, treatment planning and delivery were performed according to institutional standards, ensuring adequate coverage of the resection cavity while minimizing dose exposure to surrounding healthy brain tissue. The planning target volume (PTV) was defined as the resection cavity plus a 3-mm margin. Depending on resection status patients received either 5 × 6 Gy (R0) or 5 × 7 Gy (R1). The median PTV was 34.7 cm³ (range: 6.5–123.3 cm³), and the median V25 was 33.7 cm³.

In both groups three patients underwent simultaneous resection of two brain metastases in a singular surgical procedure. Additionally two patients in the IORT group had a second brain surgery of a distant metachronous brain metastasis in the further course of disease.

22 patients in the HSRT group and 16 patients in the IORT group had additional non-resected brain metastases. All these non-resected metastases were treated with stereotactic radiosurgery (20 Gy/ 80% Isodose) after surgery.

Post-treatment follow-up consisted of standardized contrast-enhanced magnetic resonance imaging (MRI) at three-month intervals. Follow-up assessments included evaluation of intracranial progression and treatment-related toxicities such as RN in accordance with institutional protocols [4].

### Statistical analysis

All patients included in this analysis were identified using the oncology information system MOSAIQ (ELEKTA AB, Stockholm, Sweden). Additional clinical and demographic data were retrieved from the hospital information system ORBIS (DEDALUS Healthcare Group AG, Bonn, Germany). Radiological imaging, including pre- and post-treatment assessments, was obtained from the radiology information system and picture archiving and communication system (PACS) Deep Unity (DEDALUS Healthcare Group AG, Bonn, Germany). All statistical analyses of this article were performed with statistical software ‘EZR’ (Easy R; Version 3.4.1 /The R Foundation for statistical computing). Survival outcomes were estimated using Kaplan-Meier analyses, with log-rank tests for group comparisons. A significance threshold of *p* < 0.05 was applied.

## Results

### Baseline characteristics

A total of 129 patients (HSRT: 72, IORT: 57) with 137 treated cavities (HSRT: 75, IORT: 62) were included in the analysis. The median age at treatment was 64 years in both groups. No significant differences were observed regarding recursive partitioning analysis (RPA) classification [7], metastasis size, or the number of brain metastases. However, histological distribution varied, with a higher proportion of breast cancer cases in the HSRT cohort, while NSCLC was more prevalent in the IORT group but not significantly. Prior brain irradiation was documented in 9.7% of HSRT and 10.5% of IORT patients, with a small subset having received previous irradiation to the treated area (2.8% vs. 5.3%). Detailed patient characteristics are presented in Table 1.

**Table 1:** Patient characteristics; adenocarcinoma of the esophagogastric junction (AEG), renal cell carcinoma (RCC), miscellaneous (misc. - various tumor types)

	HSRT	IORT	
**patients**	72	57	
**lesions**	75	62	
**age** (median, range)	64 years (34–87 years)	64 years (39–88 years)	*p* = 0.989
**RPA class**	1 = 10 (13.9%) 2 = 54 (75.0%) 3 = 8 (11.1%)	1 = 8 (14.0%) 2 = 41(72.0%) 3 = 8(14.0%)	*p* = 0.919
**histology**: lung (adeno) NSCLC breast cancer melanoma colorectal cancer ovarian cancer AEG RCC Misc.	20 (27.8%) 4(5.6%) 18(25.0%) 9(12.5%) 7(9.7%) 3(4.2%) 2(2.8%) 2(2.8%) 7(9.7%)	12(21.1%) 11(19.3%) 8(14.0%) 8(14.0%) 6(10.5%) 2(3.5%) 2(3.5%) 3(5.3%) 5(8.8%)	*p* = 0.306
**number of metastases** per patient (median, range)	1 (1–4)	1(1–6)	*p* = 0.767
**prior brain irradiation**	7(9.7%)	6(10.5%)	*p* = 0.735
**pre-irradiation in treated area**	2(2.8%)	3(5.3%)	*P* = 0.658

### Treatment timing, dosimetry, and surgical impact

The median time from surgery to HSRT initiation was 29 days (14–71 days), whereas IORT was administered intraoperatively. The median biologically effective dose (BED, α/β = 10) at the PTV margin (3 mm) was 48 Gy for HSRT and 50 Gy for IORT. Median operation room (OR) time was increased by 25 min in the IORT group (162 min vs. 137 min, *p* < 0.025). Further details on treatment characteristics are summarized in Table 2.

**Table 2:** Treatment characteristics

	HSRT	IORT	
**OR time** (median, range)	137 min (66–236 min)	162 min (89–308 min)	*p* = 0.025
**time from surgery to RT** (median, range)	29 days (14–71 days)	0 days	*p* < 0.001
**size of metastasis**	median: 34 mm (10–67 mm) mean: 33 mm	median: 30 mm (16–70 mm) mean: 32 mm	*p* = 0.357
median **BED** (a/ß=10) at PTV margin (3 mm)	48 Gy(48–59.5 Gy) 5 × 6 Gy(R0) 5 × 7 Gy(R1)	50 Gy(28,4–59 Gy) = 20 Gy(13,4–30 Gy) on applicator surface	*p* < 0.001
**suspected incomplete resections** on MRI	26(34.7%)	18(29.0%)	*p* = 0.378
**time to discharge** (median, range)	8 days(3–41 days)	7 days(2–41 days)	*p* = 0.207
**follow up**: MRI	median: 7.7 mo. (0-2506 days) mean: 18.2 mo.	median: 8,7 mo. (2-2614 days) mean: 15.2 mo.	*p* = 0.997

### Treatment outcomes

#### Radionecrosis rates

##### Overall incidence

**Fig. 1:**
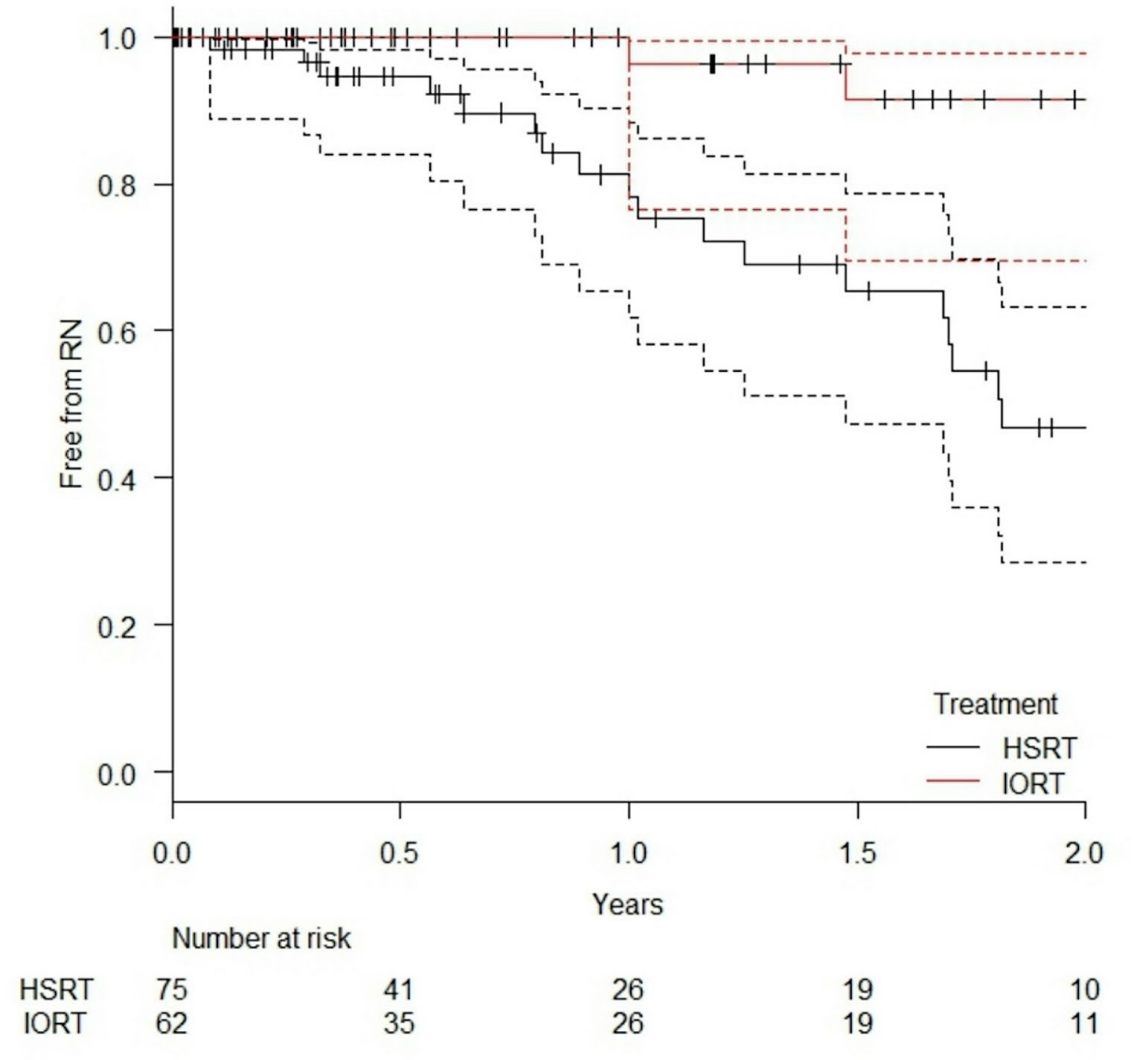
Probability of being radionecrosis-free in all treated lesions, irrespective of prior cerebral irradiation

The overall one-year incidence of RN per treated lesion was significantly lower in the IORT group, with 3.7% (95% CI: 0.5–23.5%), compared to 21.8% (95% CI: 11.7–39.2%) in the HSRT group (*p* = 0.000249). At two years, the cumulative RN incidence remained considerably lower in the IORT group at 8.5% (95% CI: 2.6–30.4%) versus 53.2% (95% CI: 36.8–71.5%) in the HSRT group. These values include both symptomatic and asymptomatic cases, regardless of prior cerebral irradiation. The Kaplan–Meier curve in Fig. 1 illustrates the probability of being RN-free over time for all treated lesions, with 95% confidence intervals.

#### Incidence in lesions without prior cerebral irradiation

**Fig. 2:**
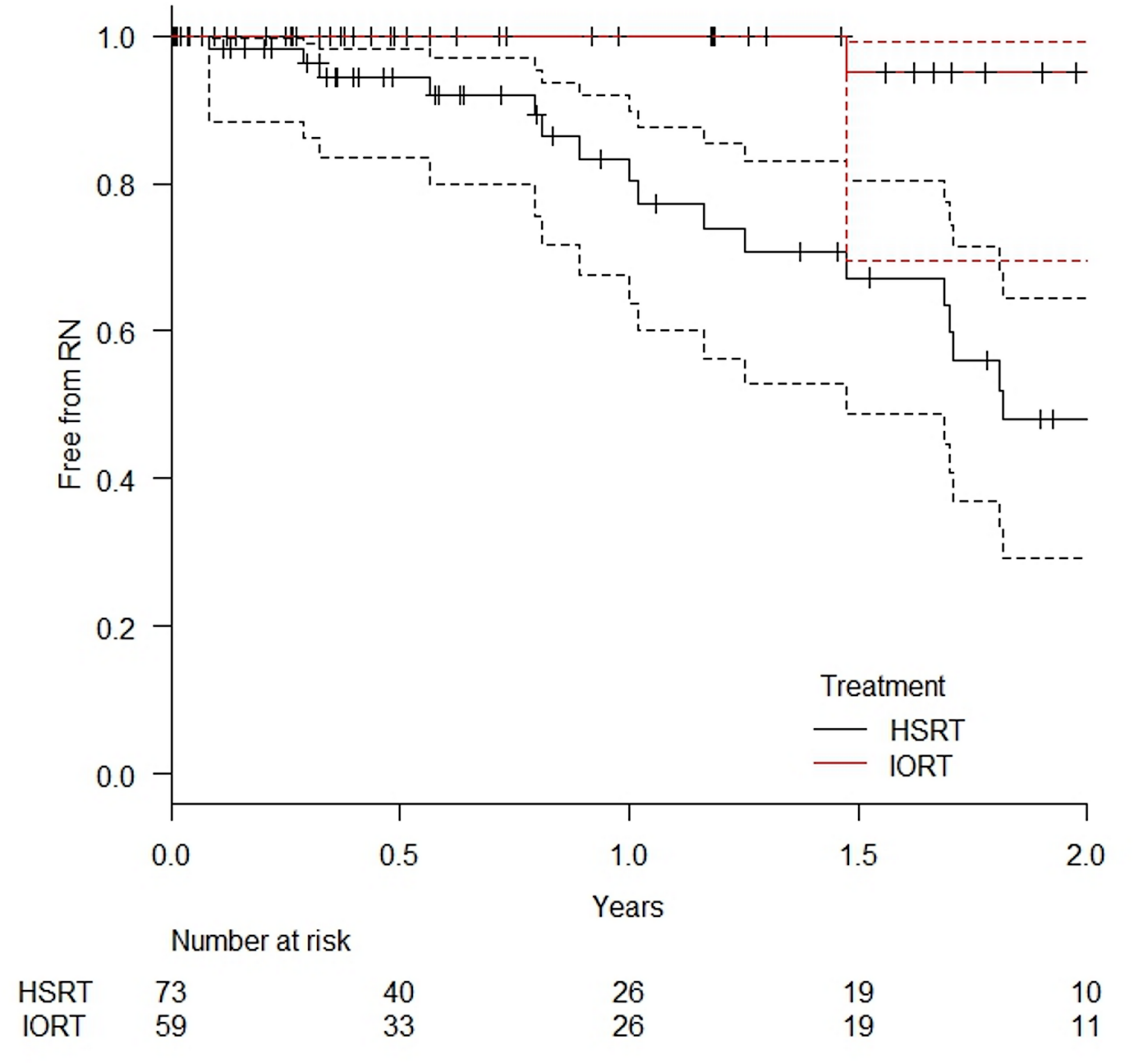
Probability of being radionecrosis-free in all treated lesions in patients without prior cerebral irradiation

In the subgroup of patients without prior cerebral irradiation, the one-year incidence of RN was 0% in the IORT group and 19.7% (95% CI: 12.0–36.2%) in the HSRT group (*p* = 0.000134). At the two-year mark, RN was observed in 5.0% (95% CI: 0.7–30.5%) of lesions treated with IORT and in 52.0% (95% CI: 37.5–80.7%) of those treated with HSRT. Figure 2 shows the corresponding RN-free survival in this subgroup.

#### Symptomatic radionecrosis

**Fig. 3:**
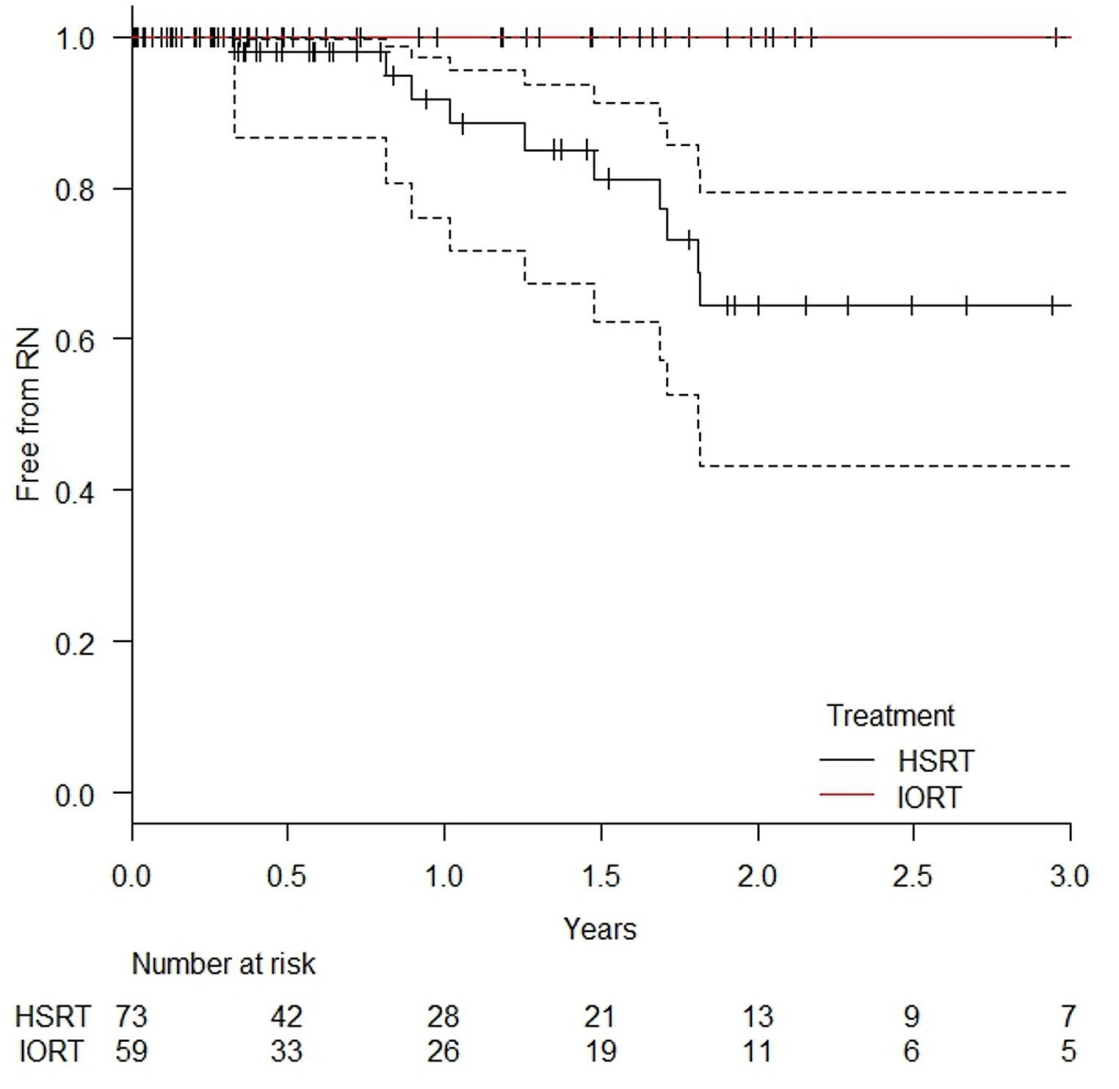
Probability of being symptomatic radionecrosis-free in all treated lesions in patients without prior cerebral irradiation

After two years, sRN occurred in 3.7% (95% CI: 0.5–23.5%) of lesions in the IORT group, compared to 35.5% (95% CI: 20.5–56.7%) in the HSRT group (*p* = 0.013). In patients without prior cerebral irradiation, no sRN cases were observed following IORT, whereas 35.5% (95% CI: 20.5–56.7%) of the HSRT-treated lesions developed sRN (*p* = 0.00362). The probability of remaining free from symptomatic RN in this subgroup is displayed in Fig. 3.

### Overall survival

**Fig. 4:**
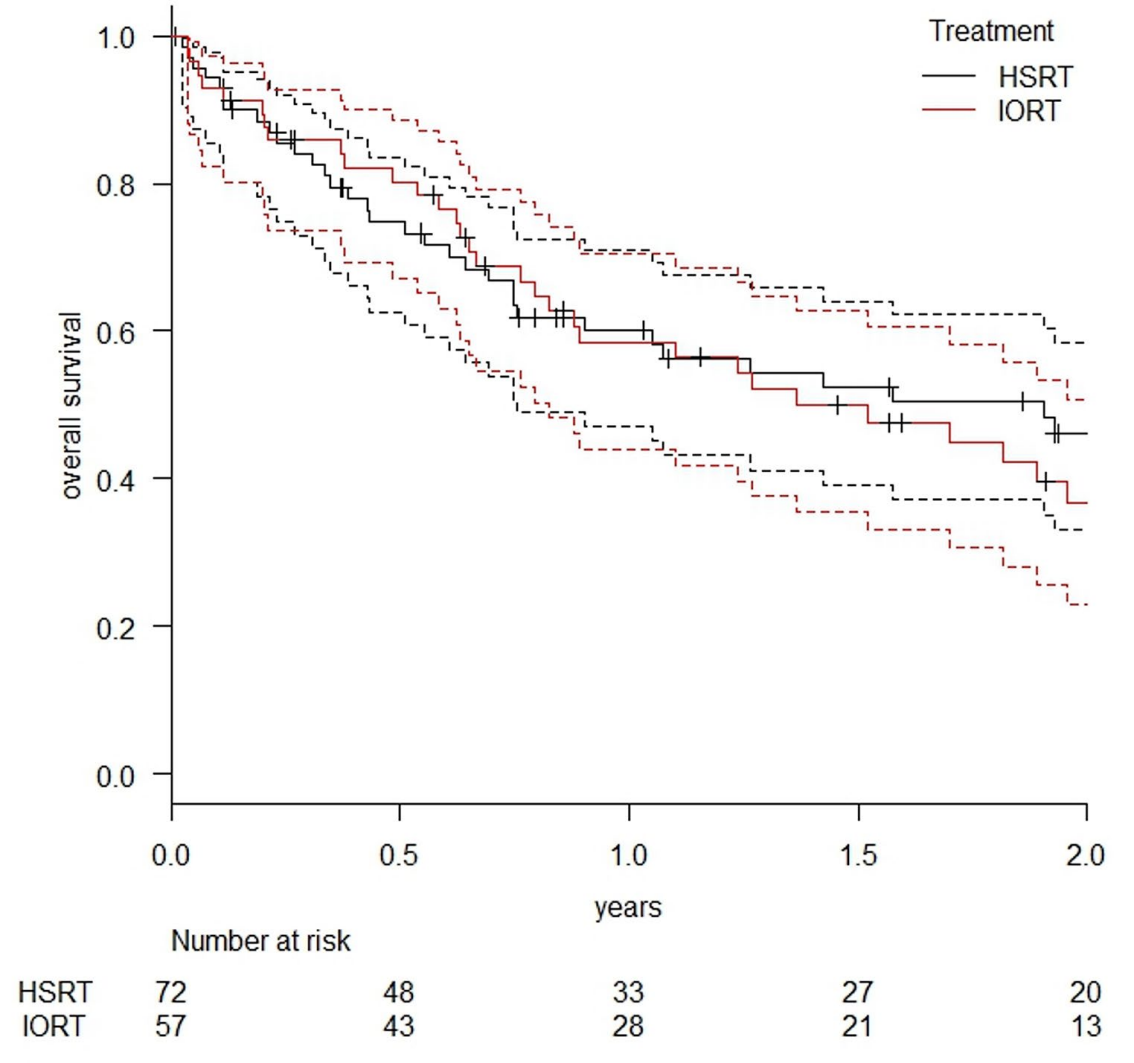
OS in patients treated with HSRT and IORT

Survival outcomes were comparable between the two cohorts, with one-year OS rates of 60.1% (95% CI: 47.0–70.9%) in the HSRT group and 58.5% (95% CI: 43.9–70.5%) in the IORT group (*p* = 0.415). The median OS reached 1.91 years (95% CI: 0.76–NA) for HSRT and 1.37 years (95% CI: 0.83–2.20) for IORT, with overlapping confidence intervals (refer to Fig. 4).

#### Intracranial progression

**Fig. 5:**
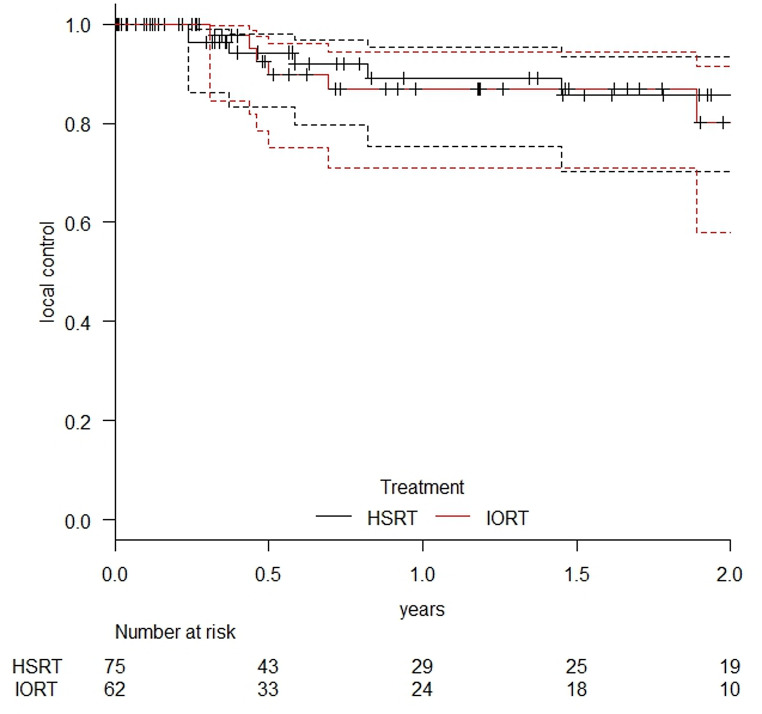
Local control rates in treated lesions after HSRT and IORT

LC at one year showed no significant difference between groups, with rates of 89.0% (95% CI: 75.2–95.4%) for HSRT and 86.8% (95% CI: 71.0–94.3%) for IORT (refer to Fig. 5). No statistically significant difference was observed (*p* = 0.706).

Similarly, intracranial disease control at one year was observed in 77.2% of patients in the HSRT group (95% CI: 62.4–86.8%) and 71.3% in the IORT group (95% CI: 54.1–83.0%), with no significant difference between groups (*p* = 0.311). Intracranial control was defined as the absence of any intracranial tumor progression as a consequence of the whole course of therapy on the most recent imaging prior to death or at last follow-up.

**Fig. 6:**
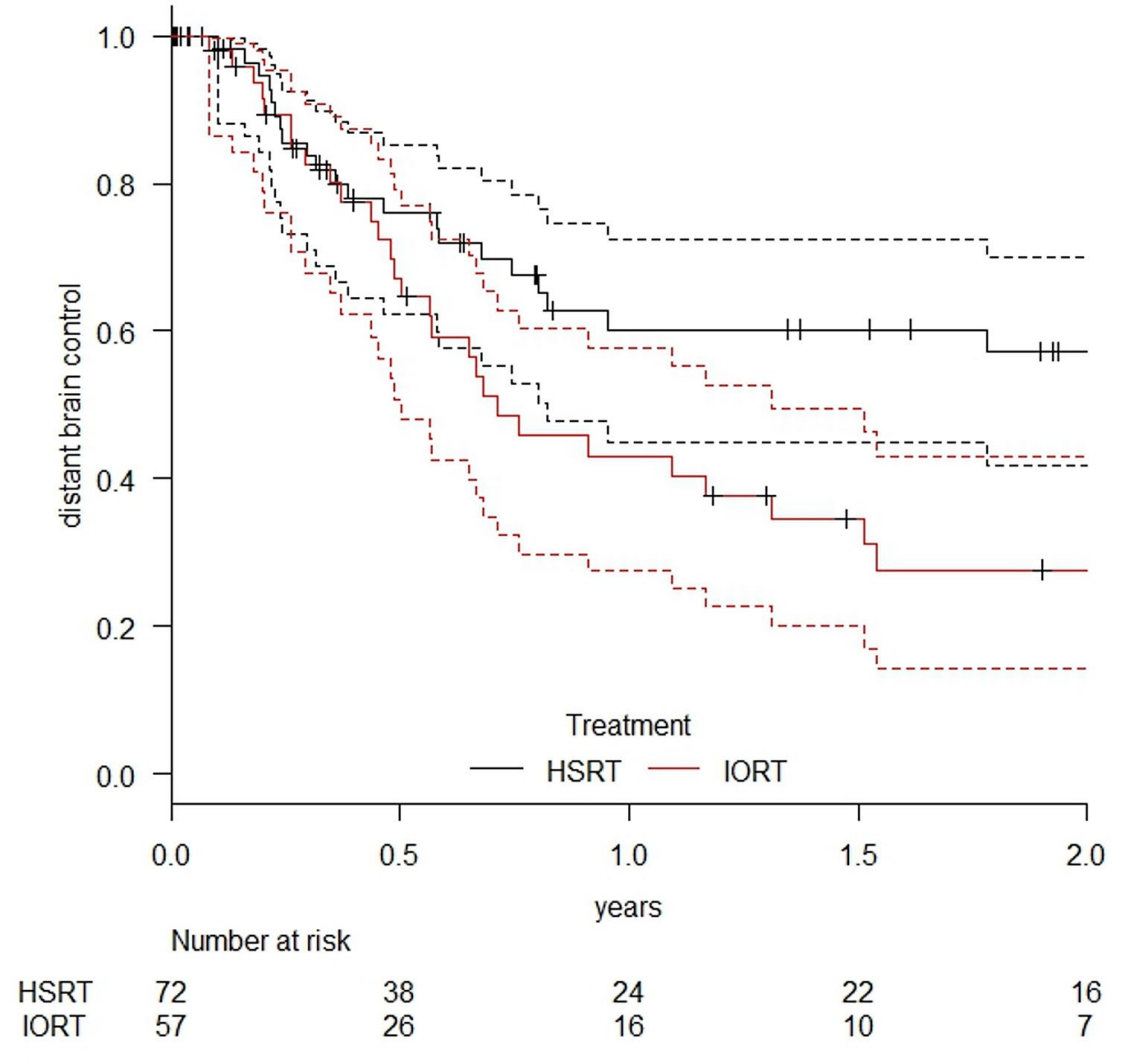
Distant brain control in patients treated with HSRT or IORT

Distant brain control at one year was significantly higher in the HSRT cohort, with 60.2% (95% CI: 45.0–72.4%) compared to 43.1% (95% CI: 27.4–57.8%) in the IORT group (*p* = 0.0167). The Kaplan-Meier curve is shown in Fig. 6.

Distant brain control at one year was significantly higher for patients with a singular brain metastasis, with 62.8% (95% CI 49.1–73.9) compared to 32.3% (16.3–49.4%) for patients with more than on brain metastasis (*p* = 0.0011).

### Leptomeningeal dissemination

**Fig. 7:**
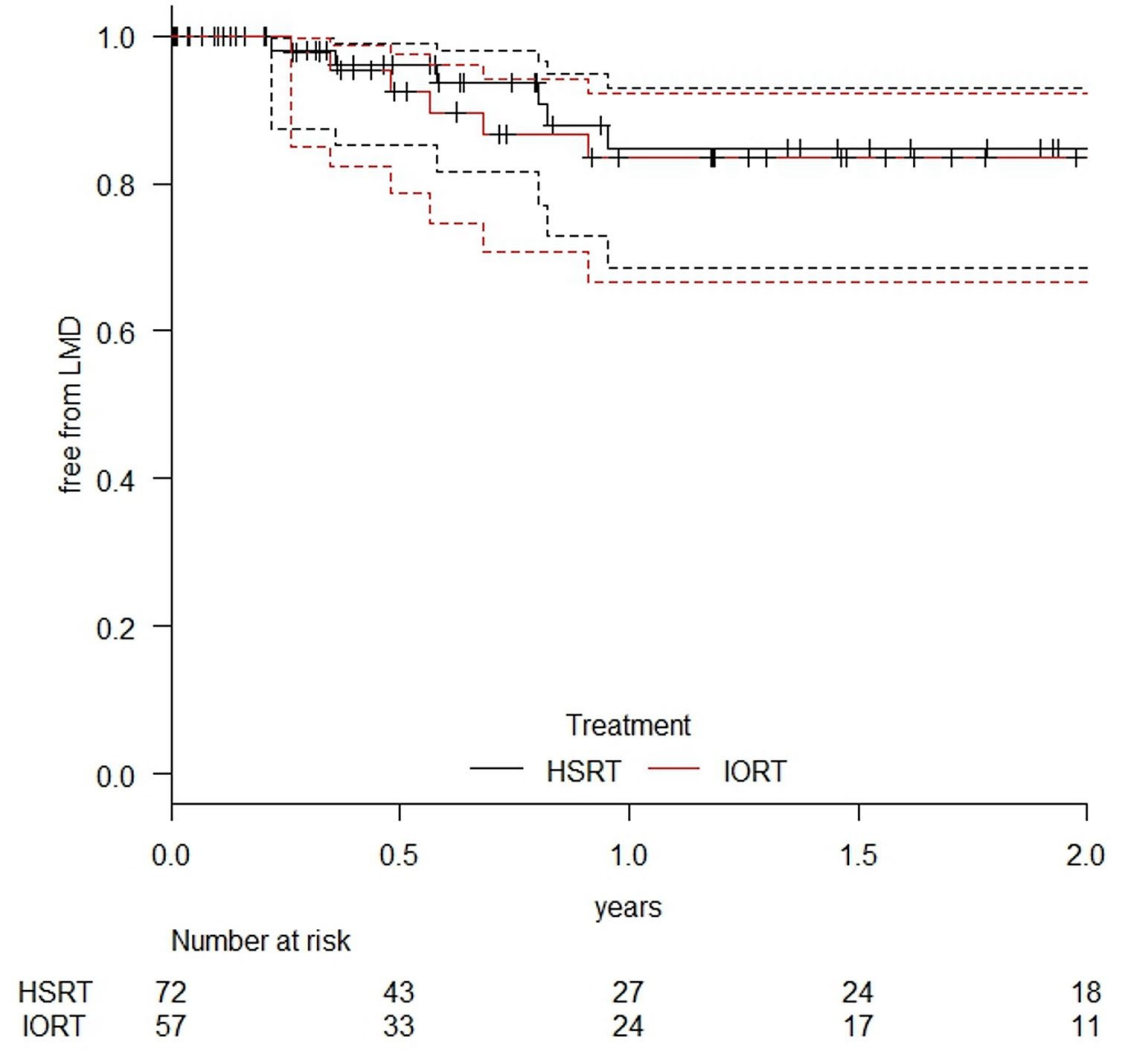
Leptomeningeal dissemination-free survival in the HSRT and IORT groups

The one-year rate of leptomeningeal dissemination was 15.3% (95% CI: 7.0–31.4%) in the HSRT group and 16.5% (95% CI: 7.7–33.4%) in the IORT group (*p* = 0.745). In both groups, leptomeningeal dissemination occurred in four cases after R0 resection and in two cases after R1 resection. Kaplan-Meier estimates for dissemination-free survival are presented in Fig. 7.

#### Avoidance of whole-brain irradiation

At one year, the proportion of patients who remained free from WBI was comparable between groups: 73.8% in the HSRT group (95% CI: 58.8–84.0%) and 73.1% in the IORT group (95% CI: 55.4–84.7%; *p* = 0.504).

A detailed summary of the results is provided in Table 3.

**Table 3:** Results

	HSRT	IORT	
1-year RN (overall)	21.8%(95% CI 11.7–39.2%)	3.7%(95% CI 0.5–23.5%)	*p* = 0.000249
2-year RN (overall)	53.2%(95% CI 36.8–71.5%)	8.5%(95% CI 2.6–30.4%)	
1-year RN without prior cerebral irradiation	19.7%(95% CI 12.0-36.2%)	0%	*p* = 0,**000134**
2-year RN without prior cerebral irradiation	52.0%(95% CI 37.5–80.7%)	5.0%(95% CI 0.7–30.5%)	
1-year sRN (overall)	8,2%(95% CI 2.7–23.9%)	3.7%(95% CI 0.5–23.5%)	*p* = 0.013
2-year sRN (overall)	35.5%(95% CI 20.5–56.7%)	3.7%(95% CI 0.5–23.5%)	
1-year sRN without prior cerebral irradiation	8,2%(95% CI 2.7–23.9%)	0%	*p* = 0.00362
2-year sRN without prior cerebral irradiation	35.5%(95% CI 20.5–56.7%)	0%	
1-year OS	60.1% (95% CI 47.0-70.9%)	58.5%(95% CI 43.9–70.5%)	*p* = 0.415
median survival time	1.91 years (95% CI 0.76-NA)	1.37 years (95% CI 0.83–2.20)	
1-year LC	89.0%(95% CI 75.2–95.4%)	86.8%(95% CI 71.0-94.3%)	*p* = 0.706
1-year intracranial disease control	77.2%(95% CI 62.4–86.8%)	71.3%(95% CI 54.1–83.0%)	*p* = 0.311
1-year DBC	60.2%(95% CI 45.0- 72.4%)	43.1%(95% CI 27.4–57.8%)	*p* = 0.0167
1-year LMD	15.3%(95% CI 7.0-31.4%)	16.5%(95% CI 7.7–33.4%)	*p* = 0.745
1-year WBI avoidance	73.8%(95% CI 58.8–84.0%)	73.1%(95% CI 55.4–84.7%)	*p* = 0.504

## Discussion

The findings of this study demonstrate that IORT achieves comparable one-year LC to HSRT, aligning with prior studies reporting 12-month LC rates between 84.2% and 97.1% [4, 8]. De Castro et al. [9] observed an LC rate of 87.5% at one year, reinforcing the effectiveness of IORT in preventing local recurrence, particularly in patients undergoing gross total resection (GTR). Cifarelli et al. [6] identified GTR as a key predictor of LC, highlighting the importance of meticulous surgical techniques to optimize IORT outcomes. In our cohort, we were not able to confirm a clear association between resection status and LC. This may be due to the presence of several cases with suspected incomplete resections due to marginal contrast enhancement around the resection cavity in the postoperative MRI that 3 months later vanished on follow up MRIs. It is likely that these represent IORT-associated radiological changes rather than residual disease. These findings underline the diagnostic challenge in differentiating between residual tumor and treatment effects, especially in the early post-IORT imaging phase.

Although previous studies suggested that IORT may reduce LMD risk, our data did not demonstrate a statistically significant difference between IORT and HSRT. Postoperative SRS cohorts have reported LMD incidences ranging from 7.2 to 28% [10, 11], while De Castro et al. [9] found no LMD cases in their IORT cohort, possibly due to immediate radiation delivery minimizing tumor cell seeding. However, differences in patient selection criteria may explain this discrepancy.

Unlike De Castro et al., who included only completely resected metastases, our study cohort also comprised cases with R1 status. But in both of our treatment groups, LMD occurred in four cases with R0 resection and in two cases with R1 resection. While an R1 status could theoretically increase the risk of LMD by leaving residual tumor cells in direct contact with the cerebrospinal fluid, our data do not indicate a clear association, as LMD also developed in R0 cases. This suggests that factors beyond the extent of resection, such as tumor biology or anatomical location, may contribute to LMD occurrence and warrant further investigation.

Appropriate patient selection remains crucial for optimizing IORT outcomes. Layer et al. [8] reported that although IORT was feasible in 88% of screened patients, it was ultimately performed in only 67%, emphasizing the need for selection criteria based on cavity geometry, tumor location, and surgical factors. However, in our cohort, all patients with a malignant intraoperative frozen section were included, without further exclusion based on anatomical constraints such as cavity location. We did not encounter technical limitations related to resection cavity geometry, apart from the predefined safety margin of 5 mm from the brainstem. These findings suggest that the feasibility of IORT may not be as restricted by anatomical considerations as previously assumed.

A major advantage of IORT is its substantially lower risk of RN compared to percutaneous techniques. In our analysis, the overall incidence of RN, including both symptomatic and asymptomatic cases, was significantly lower in the IORT group, with a one-year rate of 3.7% (95% CI: 0.5–23.5%), compared to 21.8% (95% CI: 11.7–39.2%) in the HSRT group (*p* = 0.000249). These findings were consistent over time, with a two-year cumulative incidence of 8.5% following IORT and 53.2% after HSRT. This difference was even more pronounced when considering only symptomatic RN. At the two-year mark, the Kaplan–Meier–estimated rate of symptomatic RN was 3.7% in the IORT group, in contrast to 35.5% in the HSRT group (*p* = 0.013). The observed rates of symptomatic RN were 1.6% for IORT-treated patients and 9.3% for those who underwent HSRT. These findings align with previous studies, where RN rates for EBRT range from 8% to over 20% [12, 13].

The steep dose gradient achieved with IORT likely contributes to this reduced toxicity profile, sparing adjacent healthy brain tissue more effectively than conventional techniques. A lower incidence of RN is clinically significant, as RN is associated with neurological deficits, cognitive dysfunction, and impaired quality of life. Furthermore, by reducing RN risk, IORT may decrease the need for prolonged corticosteroid therapy, thereby minimizing side effects such as weight gain, muscle atrophy, and sleep disturbances [14, 15]. Since RN often necessitates further interventions, including bevacizumab, surgical resection, or hyperbaric oxygen therapy, a lower RN rate may reduce treatment burden for patients [16]. Additionally, the lower frequency of RN may facilitate follow-up care by reducing the number of cases in which differentiating between RN and tumor recurrence poses a diagnostic challenge.

Another major benefit of IORT is its ability to expedite the initiation of systemic therapy. In a prospective study, Dejonckheere et al. [17] demonstrated a significantly shorter time to next systemic treatment (TTNT) with IORT compared to postoperative stereotactic radiotherapy (SRT), with a median TTNT of 36 days for IORT versus 52 days for SRT (*p* = 0.01). These findings were independently confirmed in a recent retrospective study from our own institution, showing that patients treated with IORT resumed systemic therapy significantly earlier than those receiving adjuvant external beam radiotherapy (32.3 ± 28.0 vs. 65.4 ± 54.3 days; *p* < 0.001) without increased postoperative morbidity [18]. Given the growing importance of timely systemic treatment, particularly for patients with rapidly progressing extracranial disease or ongoing targeted or immunotherapy, IORT may represent a practical and effective strategy to minimize treatment delays and reduce overall hospitalization time.

Despite these benefits, our data revealed a lower 1-year DBC rate in the IORT cohort compared to HSRT, consistent with De Castro et al. [9], who reported distant brain failure (DBF) rates of 50% at 6 months and 70% at 12 months. These findings suggest that while IORT effectively controls the resection cavity, it does not prevent the development of new metastatic lesions elsewhere in the brain. DBF is significantly more frequent in patients with more than one brain metastasis in both groups. This finding is consistent with SRS data showing that the risk of undetected microscopic brain metastases increases with the number of detected metastases. However, histological distribution differed between the groups, with a higher proportion of breast cancer cases in the HSRT cohort, while NSCLC was more prevalent in the IORT group. This imbalance may have influenced the observed differences in DBC, as the propensity for intracranial metastatic spread varies between tumor types. HER2-positive breast cancer, which was more common in the HSRT group, is known to respond well to systemic therapies, including HER2-targeted agents, potentially reducing the risk of new brain metastases. In contrast, NSCLC, which was more frequently observed in the IORT cohort, is associated with a higher rate of distant brain progression, particularly in cases lacking effective central nervous system (CNS)-penetrating systemic treatments.

These findings suggest that DBC may be more strongly influenced by the biology of the primary tumor rather than by the choice of local therapy. To improve intracranial disease control following IORT, future strategies should consider closer MRI surveillance and the integration of systemic agents, particularly those with CNS activity, such as immune checkpoint inhibitors in NSCLC or HER2-targeted therapies in breast cancer.

Herskind et al. [19] highlighted the potential immunomodulatory effects of high-dose irradiation, which may extend beyond direct tumor cell elimination. This raises the question of whether IORT could enhance the immune response, particularly when combined with immunotherapy. However, the observed DBF rate in the IORT cohort suggests that any potential immunomodulatory effect of IORT alone may not be sufficient to significantly impact the occurrence of new metastases. Future research should explore whether combining IORT with immune checkpoint inhibitors could enhance systemic tumor control.

Beyond oncologic and toxicity outcomes, IORT offers logistical advantages by eliminating the need for additional outpatient radiation sessions and streamlining treatment pathways into a single operative event. Prior studies have demonstrated that IORT does not increase perioperative complication rates and adds only a median of 25 min to the total operative time, further supporting its feasibility in routine clinical practice [3–5].

While our findings support the use of IORT, several limitations must be acknowledged. As a retrospective study, selection bias remains a concern, as treatment allocation was not randomized and may have been influenced by patient performance status or therapeutic preference. Additionally, differences in follow-up durations between treatment groups may have affected the observed distant control rates.

Future research should focus on optimal patient selection criteria for IORT, particularly regarding cavity size, histology, and location. The impact of IORT on neurocognitive function remains an important question, warranting further investigation in prospective studies.

Moreover, IORT has also been investigated in the treatment of glioblastoma as part of dose-escalation strategies, such as the INTRAGO study [20], which evaluates the feasibility and safety of low-energy kV X-rays for sterilizing tumor margins and modulating the postoperative microenvironment. While conceptually different from its use in brain metastases, these findings highlight the broader potential of IORT across neuro-oncological settings.

## Conclusion

Our study supports the use of IORT as a safe and effective option for adjuvant cavity irradiation, demonstrating comparable LC to HSRT while offering distinct advantages, including a significantly lower risk of RN, immediate radiation delivery, and earlier systemic therapy initiation. These benefits make IORT particularly attractive for patients requiring uninterrupted systemic treatment. However, the high rate of distant brain recurrence highlights the necessity of three-monthly MRI surveillance following focal brain radiotherapy, allowing for early detection and timely salvage treatment with SRS where applicable.

IORT and HSRT are both effective strategies for post-resection cavity irradiation. IORT may offer advantages in reducing RN and optimizing treatment sequencing, but further prospective trials are necessary to confirm these findings.

## Data Availability

The datasets generated during and/or analyzed during the current study are available from the corresponding author on reasonable request.
